# Study of large and highly stratified population datasets by combining iterative pruning principal component analysis and structure

**DOI:** 10.1186/1471-2105-12-255

**Published:** 2011-06-23

**Authors:** Tulaya Limpiti, Apichart Intarapanich, Anunchai Assawamakin, Philip J Shaw, Pongsakorn Wangkumhang, Jittima Piriyapongsa, Chumpol Ngamphiw, Sissades Tongsima

**Affiliations:** 1 Faculty of Engineering, King Mongkut's Institute of Technology Ladkrabang, Bangkok 10520, Thailand; 2 National Electronics and Computer Technology Center, Thailand Science Park, Pathumthani 12120, Thailand; 3 National Center for Genetic Engineering and Biotechnology, Thailand Science Park, Pathumthani 12120, Thailand

## Abstract

**Background:**

The ever increasing sizes of population genetic datasets pose great challenges for population structure analysis. The Tracy-Widom (TW) statistical test is widely used for detecting structure. However, it has not been adequately investigated whether the TW statistic is susceptible to type I error, especially in large, complex datasets. Non-parametric, Principal Component Analysis (PCA) based methods for resolving structure have been developed which rely on the TW test. Although PCA-based methods can resolve structure, they cannot infer ancestry. Model-based methods are still needed for ancestry analysis, but they are not suitable for large datasets. We propose a new structure analysis framework for large datasets. This includes a new heuristic for detecting structure and incorporation of the structure patterns inferred by a PCA method to complement STRUCTURE analysis.

**Results:**

A new heuristic called EigenDev for detecting population structure is presented. When tested on simulated data, this heuristic is robust to sample size. In contrast, the TW statistic was found to be susceptible to type I error, especially for large population samples. EigenDev is thus better-suited for analysis of large datasets containing many individuals, in which spurious patterns are likely to exist and could be incorrectly interpreted as population stratification. EigenDev was applied to the iterative pruning PCA (ipPCA) method, which resolves the underlying subpopulations. This subpopulation information was used to supervise STRUCTURE analysis to infer patterns of ancestry at an unprecedented level of resolution. To validate the new approach, a bovine and a large human genetic dataset (3945 individuals) were analyzed. We found new ancestry patterns consistent with the subpopulations resolved by ipPCA.

**Conclusions:**

The EigenDev heuristic is robust to sampling and is thus superior for detecting structure in large datasets. The application of EigenDev to the ipPCA algorithm improves the estimation of the number of subpopulations and the individual assignment accuracy, especially for very large and complex datasets. Furthermore, we have demonstrated that the structure resolved by this approach complements parametric analysis, allowing a much more comprehensive account of population structure. The new version of the ipPCA software with EigenDev incorporated can be downloaded from http://www4a.biotec.or.th/GI/tools/ippca.

## Background

As genotyping platforms incorporate more markers, and the costs for genotyping keep falling, ever larger and more complex datasets are being analyzed. The computationally efficient non-parametric methods for analysis of genotypic datasets are thus increasingly being used to reveal population structure. Resolution of population structure reveals evolutionary relationships between groups of individuals. Furthermore, population structure must be accounted for in genome-wide association studies to reduce spurious associations resulting from ancestral differences between cases and controls [1].

Principal component analysis (PCA) is a widely used non-parametric method for population structure analysis, which uses a covariance matrix for eigenanalysis. The amount and axes of variation among individuals are captured in the eigenvalues and eigenvectors, respectively. Previously, we developed a PCA framework for population structure analysis which extended the use of PCA beyond its usual application for visualizing the population structure trend by employing an iterative process to simplify the pattern of population structure. The iterative methods used by others, e.g. [2,3] rely on the available ethno-geographical population labels for subjectively grouping individuals, unlike our objective approach.

**Figure 1:**
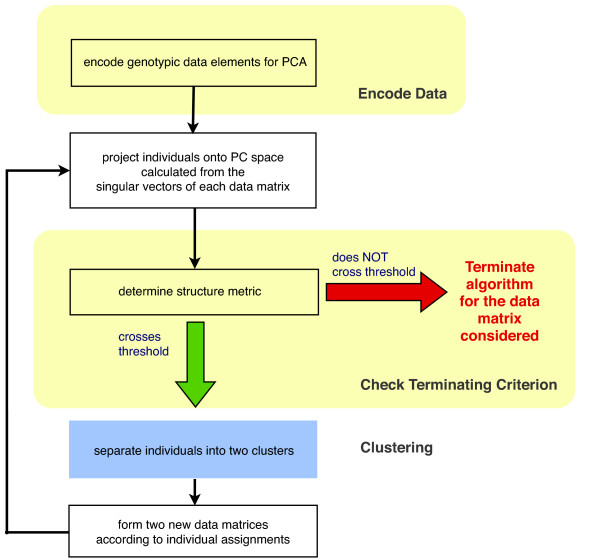
**Outline of the ipPCA framework**. The framework consists of three main components. First, the genetic data are encoded, zero-means centered and normalized. Then, individuals are projected onto a space spanned by the principal components of the input data matrix. Next, a structure metric is calculated to decide whether to advance to the clustering step or to terminate the algorithm. When the metric does not cross the threshold, a homogenous subpopulation is resolved and subsequently the algorithm terminates. Otherwise, the individuals are bisected. The algorithm iterates until all individuals have been assigned into terminal subpopulations.

Our framework, which we dubbed iterative pruning PCA (ipPCA) uses a clustering algorithm to assign individuals into subpopulations without imposing any prior assumptions [4]. ipPCA resolves all subpopulations in a population dataset, and thus reports the total number of primal subpopulations *K *in addition to assigning individuals contained within them. The term "population" is synonymous with dataset for ipPCA, which is the entire collection of individuals available for analysis. The term "subpopulation" defines a group of individuals assigned by ipPCA in which no further significant substructure is present. ipPCA operates by systematically separating individuals into two clusters using a clustering algorithm based on the Euclidean distances between projected data points and the cluster centroids. The decision to separate individuals requires testing of whether significant structure is present within the dataset (or nested dataset for subsequent iterations of the algorithm). To test for homogeneity among groups of individuals, we previously proposed using the test statistic as implemented in the EIGENSTRAT/SmartPCA algorithm, which reports the probability of structure according to Tracy-Widom (TW) distribution [5]. If no significant structure exists, then the individuals under testing belong to a subpopulation, thus terminating the iterative clustering process. The ipPCA framework is summarized in Figure 1. Using datasets of simulated and real data, we showed how ipPCA can correctly assign individuals to subpopulations and infer *K*. However, the accuracy of ipPCA may be affected by the stopping criterion. An inappropriate termination criterion leads to under- or over-estimation of the number of subpopulations. Moreover, individual assignment errors in early iterations will be compounded and carried forward to later iterations.

Parametric algorithms for clustering individuals into subpopulations, e.g., STRUCTURE, *frappe*, ADMIXTURE, and BAPS, differ from ipPCA in one crucial aspect, namely the method of assigning individuals into subpopulation clusters. The aforementioned parametric algorithms infer ancestral proportions for each individual separately, and group individuals with similar patterns of inferred ancestry. ipPCA and other non-parametric approaches cannot infer ancestry. These techniques attempt to group individuals with similar genetic profiles together. Hence, parametric approaches still offer important information not seen by non-parametric analyses. Large and highly structured population datasets are however intractable for parametric analysis because the number of *K *ancestral clusters is limited. This is due to the limited number of available samples used to estimate subpopulation allele frequencies. In order to better observe the inherent population structure, a "supervised" structure analysis, with re-sampled individuals, should be performed. The choice of individuals for such supervised analysis is arbitrary and typically guided by available ethno/geographical labels. Nonetheless, careful selection is needed to ensure that individuals being compared have similar ancestries, otherwise the signals of ancestries important for differentiating some groups of individuals may be too weak.

In this paper, we propose a modification to ipPCA by introducing a new stopping criterion called EigenDev for the iterative clustering process which is more robust to spurious patterns in large datasets. The new algorithm is termed EigenDev-ipPCA. To distinguish between the two algorithms in the ipPCA framework, we refer to the previously proposed algorithm which uses the TW statistic as the termination criterion as TW-ipPCA in the subsequent sections. Furthermore, we suggest a new protocol which uses the information from EigenDev-ipPCA to guide parametric analysis. Using real datasets, we demonstrate how this approach can reveal new and structure-informative patterns of ancestry not detectable with unsupervised STRUCTURE analysis.

## Methods

### New ipPCA terminating criterion

The Tracy-Widom (TW) test statistic, which is implemented in the EIGENSTRAT/SmartPCA algorithm [5], is used as a stopping criterion for the TW-ipPCA algorithm. Although this stopping criterion has been found to work well for some datasets, we found that when much larger datasets containing roughly >1000 individuals were analyzed, the TW-ipPCA resolved far more subpopulations than were expected. We therefore suspected that in some cases when sampling is large, the subpopulations resolved may be spurious, i.e., type I error. Indeed, as pointed out in [5], the relative sample sizes of the underlying subpopulations affect the TW test statistic.

Besides the type I error we found when using the TW statistical test for structure, there are other drawbacks which motivated us to develop an alternative terminating criterion. The first issue is computational difficulty. To obtain the final value of the TW test statistic, too many unknown parameters need to be estimated. No best estimators for these parameters are available, so choices of estimators affect the result. Instead of using the *p*-values of TW test statistics as thresholds, we propose a new terminating criterion for determining whether the data are structured. The new criterion is based on the eigenvalues of the data matrix and is termed the *EigenDev *heuristic. The EigenDev heuristic follows the same assumption as the TW theory, namely, if the first eigenvalue of the data matrix is significantly larger than the remaining eigenvalues, then substructure exists. However, we extend this observation beyond merely testing the significance of the first eigenvalue to take into account the remaining variance of the data. This allows us to observe structure in higher dimensions. We were inspired to develop EigenDev from the Eigenvalue Grads heuristic, which is applied in the signal processing domain [6]. This work showed that if the data contain only noise and no signal, i.e., non-structured, then there is an excellent linear fit for the eigenvalues ranked in descending order. In population genetic data, the noise represents the natural genetic variation within a (sub)population.

To test for population structure, the EigenDev statistic is calculated from the genotypic data. This calculation first requires that a data matrix is constructed from encoded, zero-means and normalized genotypic data, as described in [5]. This matrix contains rows corresponding to individuals and columns corresponding to alleles. Thus, biallelic SNP markers are encoded by entries in two columns, one for each allele, and STRs by the total number of alleles for that marker locus in the dataset. The presence of an allele is encoded as 1 and its absence as 0. For missing data, i.e., markers with no genotypic call, they are encoded as all 0's.

Given the zero-means, normalized genotype data matrix **X **(according to [5]) containing *m *samples with *n *allele columns per sample, we construct the sample covariance matrix

The EigenDev value can then be computed from(1)

where(2)

and(3)

where , are the first *p *eigenvalues of **C **ranked in descending order. The quantity in Eq. (1) could be negative in some cases. To militate against this possibility, the encoded entries are normalized to have zero mean. This step is important to remove the signal from the common elements, leaving only the differences (genetic variance) between individuals for eigenanalysis. In all empirical studies on both simulated and real data, we found that 90% of the variance in the data always results in a positive value and the convexity constraint in question has never been violated. To account for the rare cases when negative values are encountered, we have included a checking step in the algorithm to detect and report negative values. If negative values are found, the parameter *p *can be adjusted to ensure a positive quantity in the square root. Recall that *p *< min{*m*, *n*} is the number of eigenvalues used to compute the EigenDev statistic. We also stabilize the variance using log transformation. If the EigenDev value is large, the group of individuals being analyzed would comprise more than one subpopulation and ipPCA progresses to bisect the group; otherwise, the EigenDev-ipPCA algorithm terminates when the EigenDev value falls below a threshold.

## Results

### Testing

To test the EigenDev concept, several datasets were analyzed:

1. A simulated dataset composed of 10,000 individuals from the same population, each containing 10,000 SNP markers was used for testing the fit of TW distribution. It was generated using the GENOME tool [7] with the following parameters and the following tree file:

-pop 1 10000 -N tree.txt -C 20 -S 500

tree.txt:

0 10000

1-1

1 10000

Starting at 10,000 founder population individuals, GENOME generates the first generation with the same size as the founder. Each individual has 20 chromosomes and each chromosome contains 500 SNPs.

2. The second dataset was simulated using the same GENOME parameters as the first dataset but with different tree file:

tree.txt:

0 5000 5000

1-1 2-1

40 5000

1-1 1-2

80 5000 5000

1-1 2-1

100 10000

to generate two subpopulations of size 5,000 individuals each.

3. The third dataset is the Bovine HapMap Project collection of 497 individuals obtained from 19 different breeds, genotyped for 27203 SNPs. It is publicly available from [8].

4. The fourth dataset is publicly available from [9]. It contains 3945 individuals comprising 185 different ethno/geographical labels, typed for 1327 markers (consisting of 848 microsatellites, 476 indels, and 3 SNPs) from [10].

The ipPCA encoded input matrices from the simulated and real complex datasets are also available for download from http://www4a.biotec.or.th/GI/tools/ippca.

#### Testing metrics for population structure

**Figure 2:**
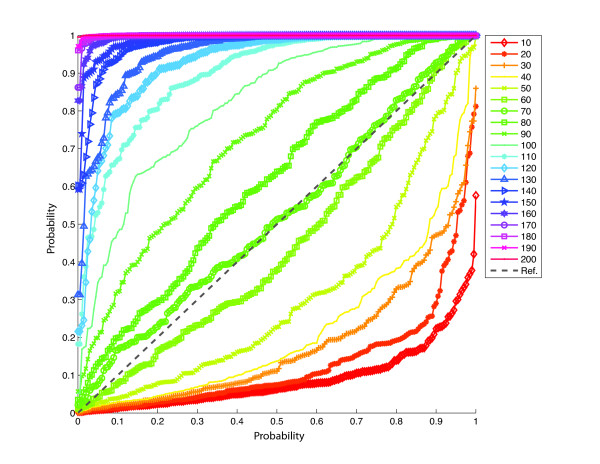
**Testing the fit of the TW distribution**. A population of size 10,000 individuals with 5,000 markers was simulated using the coalescent model. The p-p plots were generated for sample sizes of 10 to 200 individuals.

To test how TW is affected by sampling, a simulated dataset with no substructure was sampled randomly at 20 different sample sizes from 10 to 200 individuals. The corresponding probability-probability (p-p) plots for testing the fit of the TW distribution are shown in Figure 2. It is observed that the TW distribution is violated for most of the sample sizes; good fit is observed only for the sample of 70 individuals. Therefore, the deviation from TW distribution will give a false detection (type I error), particularly for large sample sizes. On the other hand, the TW test is very sensitive for detecting structure, since it is based on a non-linear phase change. It is not susceptible to type II error provided sufficient data are available [5]. However, the non-linearity of the phase change means that an all-or-nothing situation exists where the likelihood of type I cannot be controlled, even across a wide range of p-value thresholds.

**Figure 3:**
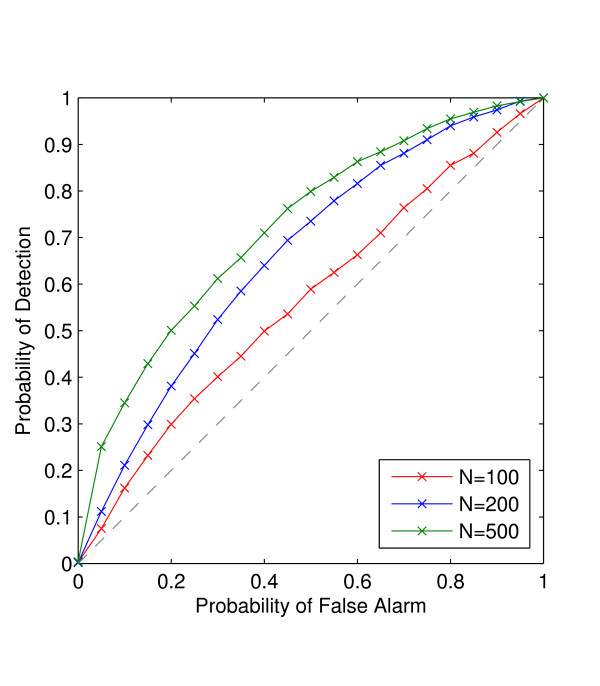
**The empirical receiver operating characteristic curve of the EigenDev heuristic**. A structured population of 10,000 individuals of 5,000 markers containing two subpopulations (5,000 each) was simulated using the coalescent model. The ROCs were generated for sample sizes of 100, 200, and 500 individuals.

To test the performance of the EigenDev heuristic, we simulated the receiver operating characteristic (ROC) curves for three different sample sizes of 100, 200, and 500 individuals from the second simulated dataset, as shown in Figure 3. To obtain the curves, the EigenDev threshold was varied between 0.077 and 0.387. It is observed that the threshold value increases with samples size, and that EigenDev performs better when the sample size is large. An EigenDev threshold of 0.21 was used for analysis of real datasets. This value is an average of the thresholds needed to achieve a 10% false positive rate for the three sample sizes. This value is a good compromise between detecting and resolving all structure present, with minimal spurious structure at typical sample sizes in real datasets.

#### Guiding parametric analysis with ipPCA

STRUCTURE [11] can be used to perform unsupervised clustering using ancestral components information. However, the high computational complexity of STRUCTURE, especially in finding the maximum posterior probabilities for the number of *K *ancestral clusters limits practically to *K *= 20 or fewer. Therefore, highly complex datasets must be divided into sub-datasets, which are then analyzed separately by STRUCTURE. Conventionally, this is done in an arbitrary fashion using prior information, e.g., ethno-geographical population labels. However, the prior information could bias the clustering results. To address this issue, we propose using the unsupervised clustering feature of ipPCA to assist in narrowing the search space for STRUCTURE in a more efficient fashion. In practice, subpopulations assigned by ipPCA can be selected for subsequent STRUCTURE analysis. We call this approach ipPCA-guided STRUCTURE. We applied this method to the Bovine HapMap dataset [8], which is the expanded dataset from the one previously analyzed by us [4]. The result was similar to that reported earlier, i.e. EigenDev-ipPCA resolved 18 subpopulations, each of which are largely composed of individuals of the same breed, except for one subpopulation containing Angus (ANG) and Red Angus (RGU) individuals (the EigenDev-ipPCA results can be viewed from the ipPCA download webpage).

**Figure 4:**
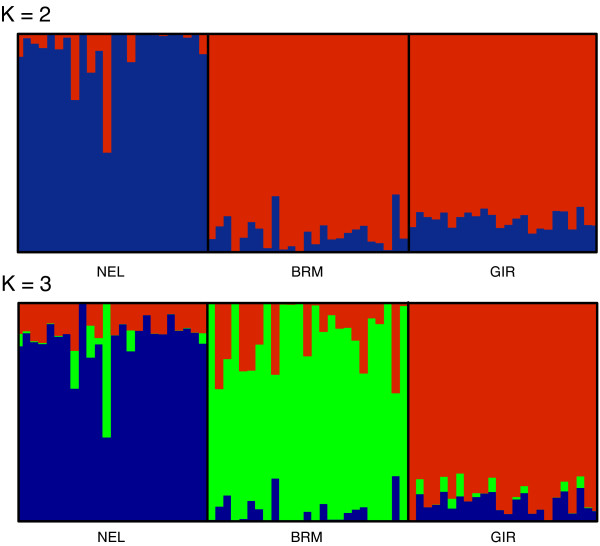
**ipPCA-guided STRUCTURE analysis on selected individuals from Bovine HapMap dataset**. STRUCTURE analyses were performed on individuals from *B. indicus *breeds (GIR, BRM, and NEL). Results with *K *= 2 and *K *= 3 are shown.

STRUCTURE was used with the default parameters and 10,000 burn in and 10,000 run iterations. Individuals from the Gir (GIR), Brahman (BRM), and Nelore (NEL) breeds resolved as three separate subpopulations by EigenDev-ipPCA) were selected for STRUCTURE analysis to determine whether differences in inferred ancestry exist between these breeds. Furthermore, these three breeds were chosen because they are *B.indicus *breeds, and thus more closely related to each other than the other *B.taurus *breeds in the dataset. STRUCTURE analysis at *K *= 3 on these selected individuals, as shown in Figure 4, revealed breed-distinctive patterns of ancestry not previously reported.

#### Analysis of a large human dataset by ipPCA

**Figure 5:**
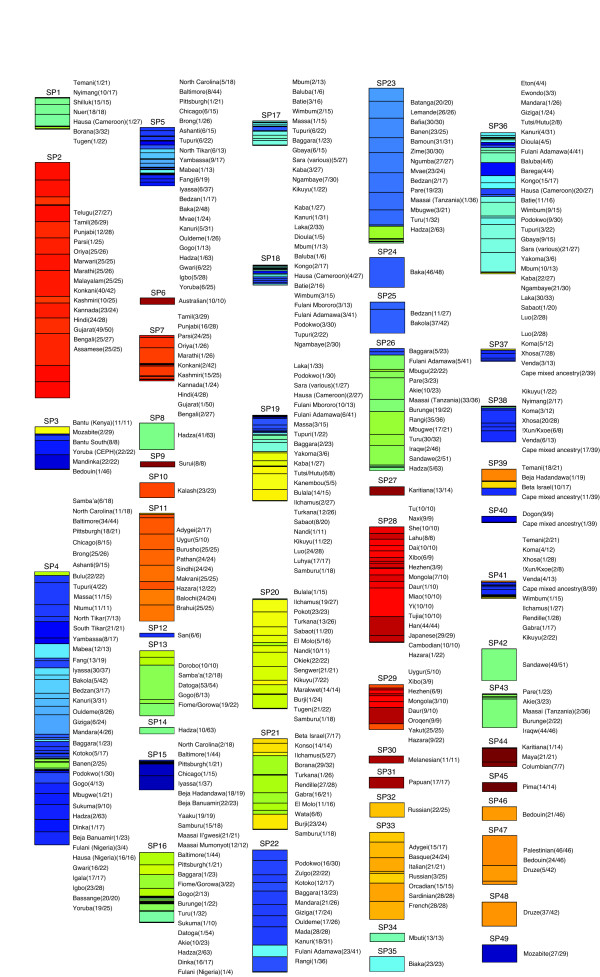
**Population assignments of the Tishkoff et al dataset using the EigenDev-ipPCA method**. 49 assigned subpopulations are labeled SP1 to SP49. The height of the bars are proportional to the number of assigned individuals in each subpopulation. The population labels of the assigned individuals are shown to the right of each bar with the number of individuals with the same label in parentheses. To aid visualization of the individual assignment, the 185 population labels were grouped into 14 color groups reflecting geographical regions. Color gradients within the color group denote different population labels. For the complete color scheme, see Figure s3 in the Additional file 1.

The dataset from Tishko et.al. [10] contains a large number of individuals (3945). Furthermore, these individuals comprise 185 ethno-linguistic distinguishing labels suggesting a large number of genetically distinct groups. The dataset was analyzed by EigenDev-ipPCA, which assigned 49 subpopulations (Figure 5). The assigned subpopulations were largely consistent with the patterns reported earlier [10], in which geographically disparate groups of individuals are genetically distinct, and within Africa, major cultural and linguistic groups are also genetically distinct (see Additional file 1 for more information). In contrast, ipPCA using the TW stopping criterion (TW-ipPCA) assigned 109 subpopulations. Comparison of the subpopulations which differed between the two methods showed that on the whole, subpopulations assigned by TW-ipPCA were sub-clusters of larger subpopulations assigned by EigenDev-ipPCA. For instance, all Indian individuals (15 ethnic labels) were assigned to two subpopulations (SP2 and SP7) by EigenDev-ipPCA, whereas Indians were assigned to 11 subpopulations by TW-ipPCA (see Additional file 1).

#### ipPCA-guided STRUCTURE analysis

African American is a term used to describe US nationals with self-identified African ancestry, the majority of whom are descended from West African individuals who came to the US via the slave trade. The term African American though is very broad, as it encompasses individuals descended from African ancestors from a broad geographical range, and some also have recent non-African ancestry. African American individuals were assigned into four subpopulations by EigenDev-ipPCA, namely SP4, SP5, SP15 and SP16. Subpopulations SP4 and SP5 contain the majority of African Americans together with predominantly West and Central African Niger-Khordofanian speaking ethnic groups. Five African Americans were assigned to SP15, which contains predominantly Afroasiatic Cushitic speaking Bejans from Sudan. Two African Americans were assigned to SP16, which contains predominantly East Africans of mixed Nilo-Saharan Sudanic and Afroasiatic Cushitic speaking ethnic groups.

**Figure 6:**
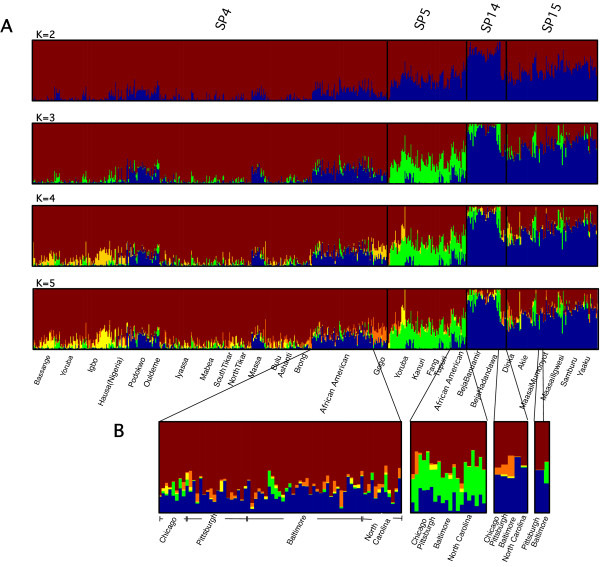
**ipPCA-guided STRUCTURE analysis on selected individuals from the Tishkoff et.al. dataset**. A) All individuals assigned to SP4, SP5, SP15 and SP16 (see Figure 5), which included all African-American individuals, were analyzed by STRUCTURE from *K *= 2 to *K *= 5. Individuals were sorted according to the ipPCA assignments. Major ethno-linguistic labels for individuals within each subpopulation are also shown (see Figure 5 for complete listing). B) Expanded view of African-American individuals from A).

We then used the information from EigenDev-ipPCA to guide STRUCTURE. All the individuals assigned to SP4, SP5, SP15 and SP16, which included all African-American individuals, were analyzed by STRUCTURE from *K *= 2 to *K *= 5 (see part A in Figure 6). At *K *= 3 or greater, each of the four subpopulations assigned by EigenDev-ipPCA showed distinctive patterns of ancestry, although there appeared to be some overlap between SP15 and SP16 individuals. When focusing on the African-American individuals, distinctive ancestry patterns can also be observed, in particular when comparing SP4 and SP5 assigned individuals (see part B in Figure 6).

## Discussion

### TW and EigenDev stopping criteria

Analysis of population genetic structure requires first a method for detecting whether significant structure exists in the dataset (or nested dataset for further iterations of ipPCA). The current method to obtain this information is to test for deviation from the Tracy-Widom distribution of the largest eigenvalue computed from PCA. A *p*-value lower than 10^-12 ^is considered an acceptable threshold for significance in rejecting the null hypothesis that the data belong to a homogenous (sub)population, and thus are structured [5]. The first experiment with a simulated dataset with no structure revealed that significant deviation from the expected distribution is found, particularly with large sampling (>70 individuals). We infer from this result that when the sample size is large, the TW method suffers from type I error because of this deviation from the TW distribution. Simply using lower *p*-value thresholds may not give better results, since there is a very small range of *p*-value that is practical [5]. When applied to real datasets, homogenous (sub)populations sampled at high density may be incorrectly construed as possessing structure. In TW-ipPCA, this would lead to a group of individuals being assigned into separate subpopulations, when they should actually be considered belonging to a single (sub)population.

To alleviate the drawbacks of the TW test statistic, we propose a new termination criterion called EigenDev statistic that is simpler to compute, has no hidden parameters and is shown to be more robust to type I error. For simplicity, one could choose a single EigenDev value to be applied as a universal stopping criterion for ipPCA, which needs to be determined empirically. We determined a threshold of 0.21 from data simulation, which was also appropriate for the real datasets analyzed in this paper.

### Analyses of Bovine HapMap dataset

The subpopulation assignment by EigenDev-ipPCA supports the accepted notion that cattle breeds have distinctive genetic profiles. The finding that ANG and RGU were assigned together in the same subpopulation suggests that these breeds are genetically indistinguishable for the markers available, which was also reported by other methods [12]. However, the finding that GIR, BRM, and NEL breeds are resolved as separate subpopulations by EigenDev-ipPCA is novel, since the earlier unsupervised STRUCTURE analysis in [12] on the entire dataset could not distinguish these breeds. ipPCA-guided STRUCTURE analysis on the Bovine HapMap dataset demonstrated differences in ancestries among these breeds, consistent with the assignments by EigenDev-ipPCA. Among these indicine breeds, there is evidence (high heterozygosity and unique SNPs) to suggest that BRM is genetically distinct from others, including GIR and NEL [12]. These results beg the question, why STRUCTURE analysis, when done in a EigenDev-ipPCA guided manner, can reveal differences among these breeds which is not apparent in the unsupervised STRUCTURE analysis? The likeliest explanation is that the overall number of informative markers is low among these indicine breeds in comparison with the others (only 19% of the loci having minor allele frequencies greater than 0.3) [12]. In other words, the allele frequencies among the indicine breeds are highly correlated in comparison with the taurine breeds. Groups of individuals with highly correlated allele frequencies in comparison with other groups tend to be merged by STRUCTURE [11].

### Analyses of a large human dataset

The 49 subpopulations assigned by EigenDev-ipPCA each contain individuals largely sharing the same ethno-linguistic label/affiliation, in accordance with [10,13]. Of note, the 426 Indian individuals were assigned to two subpopulations by EigenDev-ipPCA. This grouping is consistent with the parametric analysis of these individuals in [13], which showed weak evidence of structure. Hence, the greater degree of stratification resolved by TW-ipPCA compared with EigenDev-ipPCA is likely to be spurious. The spurious structure resolved by TW-ipPCA is thus attributable to the large sample size (426), which is well above the threshold encountered for type I error from the analysis of simulated data.

Among the African individuals, subpopulations were assigned by EigenDev-ipPCA revealing stratification patterns not described previously. For instance, Niger-Khordofanian speaking non-Pygmy individuals from West and Central Africa could not be distinguished genetically in [10], but were assigned to SP3, SP4 and SP5 subpopulations by EigenDev-ipPCA. The assignment of the majority African Americans to SP4 and SP5 by EigenDev-ipPCA (Figure 5) suggests they have West and Central African Niger-Khordofanian ancestors, in agreement with [10]. On the other hand, the assignment of African Americans to different subpopulations by EigenDev-ipPCA is suggestive of significant structure among these individuals. Supervised STRUCTURE runs performed in [10] to elucidate African American ancestry could only reveal a subtle clinal pattern of variation among the African Americans. The EigenDev-ipPCA guided STRUCTURE analysis, however, shows clear differences in ancestry between SP4 and SP5 African Americans. The SP15 and SP16 assigned African Americans also show ancestry distinct from the SP4 and SP5 assigned individuals, although given the small number of individuals assigned to SP15 and SP16, it is not possible to observe significant ancestry differences between these two groups.

The EigenDev-ipPCA assignment of some African Americans to SP15 and SP16 was unexpected. The contemporary African individuals in these subpopulations are predominantly from Saharan and East Africa. A recent study of African American ancestry concluded that some individuals have a major ancestral component which is neither West African Niger-Khordofanian, nor European [14]. The possibility that this anomalous ancestry is of Saharan or East African may also be reflected in mtDNA haplotypes, since some African Americans have anomalous haplotypes of unknown African origin [15,16]. The discrepancy between EigenDev-ipPCA guided STRUCTURE and supervised STRUCTURE performed in [10] is due to the choice of individuals in the analysis. When individuals with inappropriately diverged allele frequencies from others are used, key ancestral differences will be missed, the same as was shown in analysis of Bovine data.

## Conclusion

We describe EigenDev-ipPCA for analyzing population structure. This approach assigns individuals to subpopulations and determines the total number of subpopulations present. This algorithm incorporates a novel heuristic called EigenDev for detecting substructure, which is applied to the iterative clustering process. EigenDev is robust to population sampling, allowing us to analyze large complex datasets with higher accuracy. The subpopulations assigned by EigenDev-ipPCA reveals overall genetic relatedness among groups of individuals, which can then be used to guide STRUCTURE. Other parametric algorithms such as Admixture and *frappe *could also be used in the same way. Therefore, the combination of EigenDev-ipPCA and STRUCTURE are complementary and can be used together to perform a powerful population stratification analysis. The software both in Matlab source code (m- file) and executable versions on Windows and Linux (64 bit) are available for download at http://www4a.biotec.or.th/GI/tools/ippca.

## Competing interests

The authors declare that they have no competing interests.

## Authors' contributions

TL, AI, PJS and ST wrote the manuscript. TL, AI and ST constructed the computational improvement scheme of the new algorithm. AA, PJS and JP conceived the ideas to reanalyze the mixed complex datasets. TL, AI, PW and CN conducted all the experiments presented in this work. TL, AA, PJS, JP and ST analyzed the results. AI, PW and CN wrote the EigenDev-ipPCA program and made it available in executable formats using a Matlab compiler. All authors have read and approved the final manuscript.

## Supplementary Material

Additional file 1 **The detailed analysis and further discussion of the EigenDev-ipPCA results for the Tishkoff et al. dataset**. Click here for file
