# Supplementary material for: Study of large and highly stratified population datasets by combining iterative pruning principal component analysis and structure
Source: BMC Bioinformatics. 2011 Jun 23;12:255. doi: 10.1186/1471-2105-12-255 (PMC3148578; doi:10.1186/1471-2105-12-255)
Supplement: Additional file 1 — The detailed analysis and further discussion of the EigenDev-ipPCA results for the Tishkoff et al. dataset. [file 1471-2105-12-255-S1.PDF]

Study of large and highly stratified population datasets by combining iterative pruning principal component analysis and STRUCTURE: Supplementary Materials

Tulaya Limpiti[1], Apichart Intarapanich[2] , Anunchai Assawamakin[3] , Philip J Shaw[3] , Pongsakorn Wangkumhang[3] , Jittima Piriyapongsa[3] , Chumpol Ngamphiw[3] and Sissades Tongsima*[3]

[1]Faculty of Engineering, King Mongkut's Institute of Technology Ladkrabang, Bangkok 10520, Thailand
[2]National Electronics and Computer Technology Center, Thailand Science Park, Pathumthani 12120, Thailand
[3]National Center for Genetic Engineering and Biotechnology, Thailand Science Park, Pathumthani 12120, Thailand

Email: Tulaya Limpiti - kltulaya@kmitl.ac.th; Apichart Intarapanich - apichart.intarapanich@nectec.or.th; Anunchai Assawamakin - anunchaiice@gmail.com; Philip J Shaw - philip@biotec.or.th; Pongsakorn Wangkumhang - pongsakorn.wan@biotec.or.th; Jittima Piriyapongsa - jittima.pir@biotec.or.th; Chumpol Ngamphiw - chumpol.nga@biotec.or.th; Sissades Tongsima*- sissades@biotec.or.th;

*Corresponding author

More discussion on EigenDev-ipPCA assigned subpopulations of Tishkoff et.al. dataset

Given the complexity of population structure that is apparent in Africa, as shown in [1], detailed analysis of the EigenDev-ipPCA results for the African subpopulations is of interest.

Concordance of EigenDev-ipPCA subpopulations with model-based population clusters

Population structure clusters among Africans were identified by TESS analysis in [1]. Six clusters were resolved which reflected not only geographical proximity, but also linguistic affiliation. The largest cluster is spread over West, Central and South Africa and contains predominantly Niger-Khordofanian speaking individuals. The large extent of this genetic cluster reflects the known eastward and southward expansion of Bantu Niger-Khordofanians from Nigeria and Cameroon within the past 5000 years [2]. The inferred ancestry of the different Niger-Khordofanian ethnic groups by STRUCTURE [1] revealed substructure within this large group of individuals, reflecting admixture with local populations during the expansion across Africa.

In our analysis, the majority of African individuals were assigned by EigenDev-ipPCA to subpopulations with wide geographical and ethno-linguistic coverage, yet majority of Niger-Khordofanian speaking individuals. This is exemplified by the largest subpopulation SP4. A similar pattern to SP4 is also found among SP3, SP5 (West and Central Africa), SP17, SP18, (Central Africa), SP23, (Central and East Africa), and SP37, SP38 (East, West, Central and South Africa). These ipPCA subpopulations therefore correspond with the largest TESS cluster identified by [1], and also reflect the regional clustering caused by local admixture.

Moreover, other EigenDev-ipPCA resolved subpopulations also correspond with the other clusters resolved by TESS in [1]. These include: SP21 (Afroasiatic Cushitic speakers in East Africa); SP22 (Nilo-Saharan Chadic speakers); SP13, SP16, SP19, SP26, SP36 and SP43 (majority Afroasiatic Cushitic/Nilo-Saharan Sudanic with minority of Niger-Khordofanian speakers predominantly from East Africa and a smaller number from Central Africa); SP34 SP12, SP42 (Pygmy/Khoesan speakers); and SP8, SP14 (Hadza). The high correspondence of the ipPCA resolved African subpopulations with the results from parametric analyses therefore support the main conclusions in [1] that genetically distinct clusters exist across Africa which reflect the major linguistic divisions.

New insights into African population structure

On the other hand, more in-depth analysis of the EigenDev-ipPCA results revealed possibly new insights. The current Hadza population is small (< 1000) and lives isolated from other ethnic groups in Tanzania [3], so it is highly probable that they may appear genetically distinct because of inbreeding or drift. Indeed, the majority of Hadza individuals are assigned to two exclusively Hadza subpopulations (SP8 and 14) by ipPCA reflecting their genetic distinctiveness. On the other hand, small numbers of Hadza were also assigned by ipPCA into five other subpopulations (SP4, 5, 16, 23, 26) each of which contain a wide variety of other ethnic groups from East, West and Central Africa. The admixture pattern was missed in the analysis in [1], since the signal was masked by other ancestral components. The EigenDev-ipPCA approach reveals this pattern since it is unbiased in how individuals are compared. We interpret from the EigenDev-ipPCA pattern that the Hadza have a shared, common, and ancient ancestry with many other African peoples. This interpretation fits with the notion that the Hadza are indigenous to east Africa, from which a proto-Khoesan population diverged > 35000 years ago [1] predating the Niger-Khordofanian Bantu expansion.

The Fulani are a nomadic people with distinctive language and culture, which are spread over west and central Africa. In [1], STRUCTURE and phylogenyetic tree analysis suggested that the Fulani are genetically distinct, although mixed ancestries are apparent in STRUCTURE analysis among these individuals. EigenDev-ipPCA assigned Fulani to 5 subpopulations (SP4, 16, 18, 19, 22), however none of these were exclusively Fulani. The assigned individuals in each of these subpopulations reflect local geography, e.g. Nigerian Fulani were assigned with other Nigerians, rather than Cameroonian Fulani. Therefore, from our analysis it is clear that the Fulani are genetically heterogeneous because of local admixture, and cannot be considered as a single distinct subpopulation.

List of Supplementary Table and Figures

Table s1: Detailed individual assignments of the 185 population labels in the Tishkoff et al dataset.

The labels are grouped according to the geographical regions. The 3rd and 4th columns show subpopulation indices and the number of individuals assigned to each subpopulation (in parentheses) from the TW- and EigenDev-ipPCA algorithms. The last two columns show the number of subpopulations the individuals were assigned to for TW-ipPCA and EigenDev-ipPCA, respectively.

Figure s1: Population assignments of the Tishkoff et al dataset using TW-ipPCA for subpopulation 1 to 64.

ipPCA was performed using the TW stopping criterion on the Tishkoff et al dataset. 109 subpopulations were resolved by this anlaysis, in comparison to the 49 subpopulations by EigenDev-ipPCA. The first 64 assigned subpopulations are labeled SP1 to SP64. The height of the bars are proportional to the number of assigned individuals in each subpopulation. The population labels of the assigned individuals are shown to the right of each bar with the number of individuals with the same label in the parentheses. To aid visualization of the individual assignment, the 185 population labels were grouped into 14 color groups reflection geographical regions. Color gradients within the color group denote different population labels. For the complete color scheme, see supplementary Figure s3.

Figure s2: Population assignments of the Tishkoff et al dataset using TW-ipPCA for subpopulations 65 to 109.

The assigned subpopulations are labeled SP65 to SP109, which is the continuation of Figure s1.

Figure s3: Color scheme for the 185 population labels in the Tishkoff et.al. dataset

.

Table s1:

Labels	Region	Subpopulation assignments		Number of groups	
		TW	EigenDev	TW	EigenDev
Baltimore	African American	14(1), 15(1), 39(3), 40(4), 41(1), 65(31), 67(1), 68(1), 71(1)	4(34), 5(8), 15(1), 16(1)	9	4
Chicago	African American	15(1), 40(6), 65(8)	4(8), 5(6), 15(1)	3	3
North Carolina	African American	15(2), 39(2), 40(3), 65(9), 68(1), 103(1)	4(11), 5(5), 15(2)	6	3
Pittsburgh	African American	15(1), 40(1), 41(1), 65(17), 71(1)	4(18), 5(1), 15(1), 16(1)	5	4
Columbian	Americas	20(7)	44(7)	1	1
Karitiana	Americas	11(13), 20(1)	27(13), 44(1)	2	2
Maya	Americas	20(21)	44(21)	1	1
Pima	Americas	21(14)	45(14)	1	1
Surui	Americas	11(8)	9(8)	1	1
Australian	Australia	13(9), 63(1)	6(10)	2	1
Adamawa	Central Africa	29(3), 47(12), 48(9), 49(1), 53(5), 60(4), 86(1), 95(3), 96(3)	18(3), 19(6), 22(23), 26(5), 36(4)	9	5
Bafia	Central Africa	50(1), 51(15), 52(13), 88(1)	23(30)	4	1
Baggara	Central Africa	30(1), 41(1), 48(13), 53(4), 69(1), 95(2), 108(1)	4(1), 16(1), 17(1), 19(2), 22(13), 26(5)	7	6
Baka	Central Africa	12(46), 14(2)	5(2), 24(46)	2	2
Bakola	Central Africa	25(37), 66(5)	4(5), 25(37)	2	2
Baluba	Central Africa	5(1), 29(1), 96(1), 97(2), 98(1)	17(1), 18(1), 36(4)	5	3
Bamoun	Central Africa	50(1), 51(17), 52(13)	23(31)	3	1
Banen	Central Africa	51(16), 52(7), 102(1), 103(1)	4(2), 23(23)	4	2
Barega	Central Africa	94(1), 96(1), 97(2)	36(4)	3	1
Batanga	Central Africa	50(13), 51(3), 52(3), 87(1)	23(20)	4	1
Batie	Central Africa	29(2), 30(3), 96(1), 97(7), 99(3)	17(3), 18(2), 36(11)	5	3
Bedzan	Central Africa	14(1), 24(11), 66(3), 87(2)	4(3), 5(1), 23(2), 25(11)	4	4
Biaka	Central Africa	9(23)	35(23)	1	1
Bulala	Central Africa	30(1), 91(2), 95(12)	19(14), 20(1)	3	2
Bulu	Central Africa	66(6), 67(2), 68(2), 102(7), 103(5)	4(22)	5	1
Dinka	Central Africa	41(1), 72(16)	4(1), 16(16)	2	2
Eton	Central Africa	94(1), 96(2), 97(1)	36(4)	3	1
Ewondo	Central Africa	97(3)	36(3)	1	1
Fang	Central Africa	14(6), 66(4), 102(3), 103(6)	4(13), 5(6)	4	2
Gbaya	Central Africa	30(6), 94(1), 95(1), 96(4), 97(2), 99(1)	17(6), 36(9)	6	2
Giziga	Central Africa	41(6), 48(13), 49(4), 94(1)	4(6), 22(17), 36(1)	4	3
Hausa (Cameroon)	Central Africa	2(1), 29(4), 94(5), 95(3), 96(2), 97(8), 99(4)	1(1), 18(4), 19(2), 36(20)	7	4
Iyassa	Central Africa	14(3), 15(1), 39(3), 66(15), 67(5), 102(3), 103(7)	4(30), 5(6), 15(1)	7	3
Kaba	Central Africa	29(1), 30(3), 94(17), 96(1), 97(1), 98(2), 99(2)	17(3), 18(1), 19(1), 36(22)	7	4
Kanembou	Central Africa	91(1), 95(3), 99(1)	19(5)	3	1
Kanuri	Central Africa	14(2), 29(1), 39(3), 41(3), 47(1), 48(13), 86(4), 95(1), 96(3)	4(3), 5(5), 18(1), 22(18), 36(4)	9	5
Kongo	Central Africa	29(2), 96(6), 97(5), 99(4)	18(2), 36(15)	4	2
Kotoko	Central Africa	41(5), 48(10), 49(2)	4(5), 22(12)	3	2
Laka	Central Africa	29(1), 94(20), 95(4), 96(3), 97(1), 98(4)	18(2), 19(1), 36(30)	6	3
Lemande	Central Africa	50(1), 51(6), 52(19)	23(26)	3	1
Mabea	Central Africa	14(1), 66(4), 68(1), 102(2) , 103(5)	4(12), 5(1)	5	2
Mada	Central Africa	48(1), 49(6), 86(21)	22(28)	3	1
Mandara	Central Africa	41(4), 48(15), 49(5), 85(1), 95(1)	4(4), 22(21), 36(1)	5	3
Massa	Central Africa	30(1), 41(10), 65(1), 95(3)	4(11), 17(1), 19(3)	4	3
Mbororo Fulani	Central Africa	29(3), 60(9), 97(1)	18(3), 19(10)	3	2
Mbum	Central Africa	29(1), 30(2), 94(6), 97(1), 98(2), 99(1)	17(2), 18(1), 36(10)	6	3
Mbuti	Central Africa	8(13)	34(13)	1	1
Mvae	Central Africa	14(1), 50(16), 51(4), 87(2), 88(1)	5(1), 23(23)	5	2
Ngambaye	Central Africa	29(2), 30(7), 94(9), 95(8), 98(3), 99(1)	17(7), 18(2), 36(21)	6	3
Ngumba	Central Africa	50(9), 51(1), 87(4), 88(13)	23(27)	4	1
North Tikar	Central Africa	14(5), 39(1), 66(1), 67(2), 103(4)	4(7), 5(6)	5	2
Ntumu	Central Africa	66(3), 68(1), 102(4), 103(3)	4(11)	4	1
Nuer	Central Africa	2(2), 3(16)	1(18)	2	1
Nyimang	Central Africa	2(8), 3(2), 34(2)	1(10), 38(2)	3	2
Ouldeme	Central Africa	14(1), 41(8), 48(2), 49(14), 85(1)	4(8), 5(1), 22(17)	5	3

Labels	Region	Subpopulation assignments		Number of groups	
		TW	EigenDev	TW	EigenDev
Podokwo	Central Africa	29(3), 41(1), 48(1), 49(15), 95(6), 96(1), 98(3)	4(1), 18(3), 19(1), 22(16), 36(9)	7	5
Sara	Central Africa	30(5), 94(4), 95(5), 96(4), 97(3), 98(5), 99(1)	17(5), 19(1), 36(21)	7	3
Shilluk	Central Africa	2(3), 3(12)	1(15)	2	1
South Tikar	Central Africa	66(13), 102(4), 103(4)	4(21)	3	1
Tupuri	Central Africa	14(5), 29(2), 30(6), 39(1), 41(4), 94(1), 95(3)	4(4), 5(6), 17(6), 18(2), 19(1), 36(3)	7	6
Tutsi/Hutu	Central Africa	91(1), 96(1), 97(2), 99(4)	19(6), 36(2)	4	2
Wimbum	Central Africa	5(1), 29(3), 30(2), 96(1), 97(8)	17(2), 18(3), 36(9), 41(1)	5	4
Yakoma	Central Africa	95(1), 96(1), 98(1), 99(3)	19(3), 36(3)	4	2
Yambassa	Central Africa	14(9), 103(8)	4(8), 5(9)	2	2
Zime	Central Africa	50(27), 87(1), 88(2)	23(30)	3	1
Zulgo	Central Africa	49(1), 85(19), 86(2)	22(22)	3	1
Balochi	Central Asia	17(19), 74(2), 75(3)	11(24)	3	1
Brahui	Central Asia	17(25)	11(25)	1	1
Burusho	Central Asia	43(25)	11(25)	1	1
Hazara	Central Asia	43(3), 74(1), 75(4), 80(13), 81(1)	11(12), 28(1), 29(9)	5	3
Kalash	Central Asia	10(23)	10(23)	1	1
Makrani	Central Asia	17(17), 74(4), 75(4)	11(25)	3	1
Pathan	Central Asia	17(2), 43(2), 74(14), 75(6)	11(24)	4	1
Sindhi	Central Asia	17(1), 74(17), 75(6)	11(24)	3	1
Uygur	Central Asia	75(3), 80(7)	11(5), 29(5)	2	2
Cambodian	East Asia	83(2), 84(8)	28(10)	2	1
Dai	East Asia	83(10)	28(10)	1	1
Daur	East Asia	79(9), 82(1)	28(1), 29(9)	2	2
Han	East Asia	81(23), 82(3), 83(18)	28(44)	3	1
Hezhen	East Asia	79(6), 81(2), 83(1)	28(3), 29(6)	3	2
Japanese	East Asia	82(29)	28(29)	1	1
Lahu	East Asia	84(8)	28(8)	1	1
Miao	East Asia	81(3), 83(7)	28(10)	2	1
Mongola	East Asia	79(3), 81(6), 82(1)	28(7), 29(3)	3	2
Naxi	East Asia	81(8), 84(1)	28(9)	2	1
Oroqen	East Asia	79(9)	29(9)	1	1
She	East Asia	81(1), 83(9)	28(10)	2	1
Tu	East Asia	81(9), 83(1)	28(10)	2	1
Tujia	East Asia	81(6), 82(1), 83(3)	28(10)	3	1
Xibo	East Asia	79(3), 81(3), 82(3)	28(6), 29(3)	3	2
Yakut	East Asia	46(24), 79(1)	29(25)	2	1
Yi	East Asia	81(9), 82(1)	28(10)	2	1
Akie	Eastern Africa	27(3), 53(10), 71(10)	16(10), 26(10), 43(3)	3	3
Beta Israel	Eastern Africa	31(16), 32(1)	21(7), 39(10)	2	2
Borana	Eastern Africa	2(3), 57(12), 58(4), 92(11), 93(2)	1(3), 21(29)	5	2
Burji	Eastern Africa	57(22), 58(2)	20(1), 21(23)	2	2
Burunge	Eastern Africa	27(3), 53(2), 71(1), 89(4), 108(3), 109(9)	16(1), 26(19), 43(2)	6	3
Datog	Eastern Africa	42(11), 69(1), 73(33), 104(4), 105(5)	13(53), 16(1)	5	2
Dorobo	Eastern Africa	42(4), 73(2), 105(4)	13(10)	3	1
El Molo	Eastern Africa	57(2), 59(8), 90(1), 91(5)	20(5), 21(11)	4	2
Fiome	Eastern Africa	42(5), 71(3), 73(13), 105(1)	13(19), 16(3)	4	2
Gabra	Eastern Africa	5(1), 31(3), 57(1), 58(4), 92(5), 93(3)	21(16), 41(1)	6	2
Gogo	Eastern Africa	39(1), 42(4), 64(3), 65(1), 71(2), 105(2)	4(4), 5(1), 13(6), 16(2)	6	4
Hadza	Eastern Africa	4(41), 16(10), 40(1), 50(1), 53(1), 64(2), 71(2), 88(1), 89(2), 108(1), 109(1)	4(2), 5(1), 8(41), 14(10), 16(2), 23(2), 26(5)	11	7
Il'gwesi Maasai	Eastern Africa	69(5), 70(8), 71(8)	16(21)	3	1
Iraqw	Eastern Africa	27(46)	26(2), 43(44)	1	2
Kenya	Eastern Africa	1(11)	3(11)	1	1
Kikuyu	Eastern Africa	5(2), 30(1), 34(1), 90(13), 95(2), 99(3)	17(1), 19(11), 20(7), 38(1), 41(2)	6	5
Konso	Eastern Africa	57(11), 58(1), 92(2)	21(14)	3	1
Luhya	Eastern Africa	30(1), 94(1), 96(6), 97(1), 99(8)	19(17)	5	1
Luo	Eastern Africa	96(12), 97(3), 99(13)	19(24), 36(2), 37(2)	3	3

Labels	Region	Subpopulation assignments		Number of groups	
		TW	EigenDev	TW	EigenDev
Maasai	Eastern Africa	27(2), 50(1), 53(28), 89(2), 108(1), 109(2)	23(1), 26(33), 43(2)	6	3
Maasai Ilchamus	Eastern Africa	5(1), 55(2), 56(2), 58(1), 91(17), 92(3), 97(1)	19(2), 20(19), 21(5), 41(1)	7	4
Marakwet	Eastern Africa	55(1), 56(12), 90(1)	20(14)	3	1
Mbugu	Eastern Africa	26(22)	26(22)	1	1
Mbugwe	Eastern Africa	50(1), 65(1), 87(1), 88(1), 89(16), 108(1)	4(1), 23(3), 26(17)	6	3
Mumonyot Maasai	Eastern Africa	69(7), 71(5)	16(12)	2	1
Nandi	Eastern Africa	30(1), 54(1), 55(1), 56(3), 90(5)	19(1), 20(10)	5	2
Okiek	Eastern Africa	54(21), 56(1)	20(22)	2	1
Pare	Eastern Africa	27(1), 50(4), 87(15), 89(2), 109(1)	23(19), 26(3), 43(1)	5	3
Pokot	Eastern Africa	55(1), 56(21), 91(1)	20(23)	3	1
Rangi	Eastern Africa	48(1), 53(1), 89(19), 108(10), 109(5)	22(1), 26(35)	5	2
Rendille	Eastern Africa	5(1), 92(3), 93(24)	21(27), 41(1)	3	2
Sabaot	Eastern Africa	55(6), 56(4), 90(3), 91(2), 95(1), 96(2), 97(1), 99(1)	19(8), 20(11), 36(1)	8	3
Samba'a	Eastern Africa	42(1), 64(6), 105(11)	4(6), 13(12)	3	2
Samburu	Eastern Africa	58(1), 69(6), 71(9), 90(1), 91(1)	16(15), 19(1), 20(1), 21(1)	5	4
Sandawe	Eastern Africa	28(48), 53(2), 89(1)	26(2), 42(49)	3	2
Sengwer	Eastern Africa	55(18), 56(3)	20(21)	2	1
Sukuma	Eastern Africa	64(7), 66(1), 69(1), 102(1)	4(9), 16(1)	4	2
Tugen	Eastern Africa	2(1), 55(19), 91(2)	1(1), 20(21)	3	2
Turkana	Eastern Africa	55(1), 56(1), 90(3), 91(21)	19(12), 20(13), 21(1)	4	3
Turu	Eastern Africa	28(1), 50(1), 53(10), 71(1), 89(7), 108(9), 109(3)	16(1), 23(1), 26(30)	7	3
Wata	Eastern Africa	58(6)	21(6)	1	1
Yaaku	Eastern Africa	69(17), 70(1), 71(1)	16(19)	3	1
Adygei	Europe	75(2), 77(5), 78(10)	11(2), 33(15)	3	2
Basque	Europe	45(24)	33(24)	1	1
French	Europe	77(2), 78(26)	33(28)	2	1
Italian	Europe	77(17), 78(4)	33(21)	2	1
Orcadian	Europe	78(15)	33(15)	1	1
Russian	Europe	19(22), 78(3)	32(22), 33(3)	2	2
Sardinian	Europe	77(28)	33(28)	1	1
Assamese	India	7(7), 37(1), 38(8), 62(8), 63(1)	2(25)	5	1
Bengali	India	7(12), 35(2), 37(3), 38(4), 62(2), 63(3), 100(1)	2(25), 7(2)	7	2
Gujarat	India	7(12), 35(2), 36(31), 37(1), 38(1), 61(1), 62(1), 100(1)	2(49), 7(1)	8	2
Hindi	India	7(13), 35(2), 37(3), 38(3), 61(2), 62(1), 63(1), 100(1), 101(2)	2(24), 7(4)	9	2
Kannada	India	7(19), 35(2), 38(1), 63(1), 100(1)	2(23), 7(1)	5	2
Kashmiri	India	7(1), 35(1), 37(12), 38(1), 61(6), 100(1), 101(3)	2(10), 7(15)	7	2
Konkani	India	7(37), 36(1), 37(3), 61(1)	2(40), 7(2)	4	2
Malayalam	India	7(11), 35(2), 37(2), 38(2), 61(2), 62(4), 100(1), 101(1)	2(25)	8	1
Marathi	India	7(12), 35(3), 37(2), 61(6), 63(2), 100(1)	2(25), 7(1)	6	2
Marwari	India	7(19), 35(1), 37(1), 38(1), 62(1), 100(1), 101(1)	2(25)	7	1
Oriya	India	7(11), 38(3), 62(4), 63(6), 100(2)	2(25), 7(1)	5	2
Parsi	India	6(24), 35(1)	2(1), 7(24)	2	2
Punjabi	India	7(3), 35(1), 37(11), 38(2), 61(3), 62(1), 63(1), 100(3), 101(3)	2(12), 7(16)	9	2
Tamil	India	7(11), 35(5), 37(3), 38(4), 61(1), 63(1), 100(3), 101(1)	2(26), 7(3)	8	2
Telugu	India	7(14), 35(2), 37(1), 38(9), 61(1)	2(27)	5	1
Bedouin	Middle East	1(1), 76(21), 107(24)	3(1), 46(21), 47(24)	3	3
Druze	Middle East	44(39), 107(3)	47(5), 48(37)	2	2
Palestinian	Middle East	106(28), 107(18)	47(46)	2	1
Melanesian	Oceania	22(11)	30(11)	1	1
Papuan	Oceania	23(17)	31(17)	1	1
Banuamir Beja	Saharan Africa	15(22), 41(1)	4(1), 15(22)	2	2
Hadandawa Beja	Saharan Africa	15(18), 31(1)	15(18), 39(1)	2	2
Mozabite	Saharan Africa	1(1), 18(28)	3(2), 49(27)	2	2

Labels	Region	Subpopulation assignments		Number of groups	
		TW	EigenDev	TW	EigenDev
!Xum/Khoe	Southern Africa	5(2), 34(6)	38(6), 41(2)	2	2
Cape Mixed Ancestry	Southern Africa	5(10), 31(1), 32(13), 33(3), 34(12)	37(2), 38(17), 39(11), 40(1), 41(8)	5	5
San	Southern Africa	8(6)	12(6)	1	1
South Bantu	Southern Africa	1(8)	3(8)	1	1
Venda	Southern Africa	5(5), 33(2), 34(5), 97(1)	37(3), 38(6), 41(4)	4	3
Xhosa	Southern Africa	5(1), 33(24), 34(3)	37(7), 38(20), 41(1)	3	3
Ashanti	Western Africa	40(6), 65(3), 67(2), 68(3), 103(1)	4(9), 5(6)	5	2
Bassange	Western Africa	65(4), 67(11), 68(5)	4(20)	3	1
Brong	Western Africa	40(1), 65(14), 67(3), 68(8)	4(25), 5(1)	4	2
Dioula	Western Africa	29(1), 96(1), 97(1), 98(2)	18(1), 36(4)	4	2
Dogon	Western Africa	5(9)	40(9)	1	1
Fulani	Western Africa	41(3), 71(1)	4(3), 16(1)	2	2
Gwari	Western Africa	39(2), 40(4), 41(1), 65(1), 67(11), 68(3)	4(16), 5(6)	6	2
Hausa (Nigeria)	Western Africa	41(2), 65(1), 67(9), 68(4)	4(16)	4	1
Igala	Western Africa	65(1), 66(1), 67(7), 68(8)	4(17)	4	1
Igbo	Western Africa	39(4), 40(1), 65(1), 67(11), 68(10), 102(1)	4(23), 5(5)	6	2
Koma	Western Africa	5(5), 34(5), 94(1), 97(1)	37(5), 38(3), 41(4)	4	3
Mandinka	Western Africa	1(22)	3(22)	1	1
Yoruba	Western Africa	14(4), 39(1), 40(1), 65(2), 66(1), 67(5), 68(10), 103(1)	4(19), 5(6)	8	2
Yoruba (CEPH)	Western Africa	1(22)	3(22)	1	1
Temani	Yemen	2(1), 5(3), 31(16), 31(1)	1(1), 39(18), 41(2)	4	3

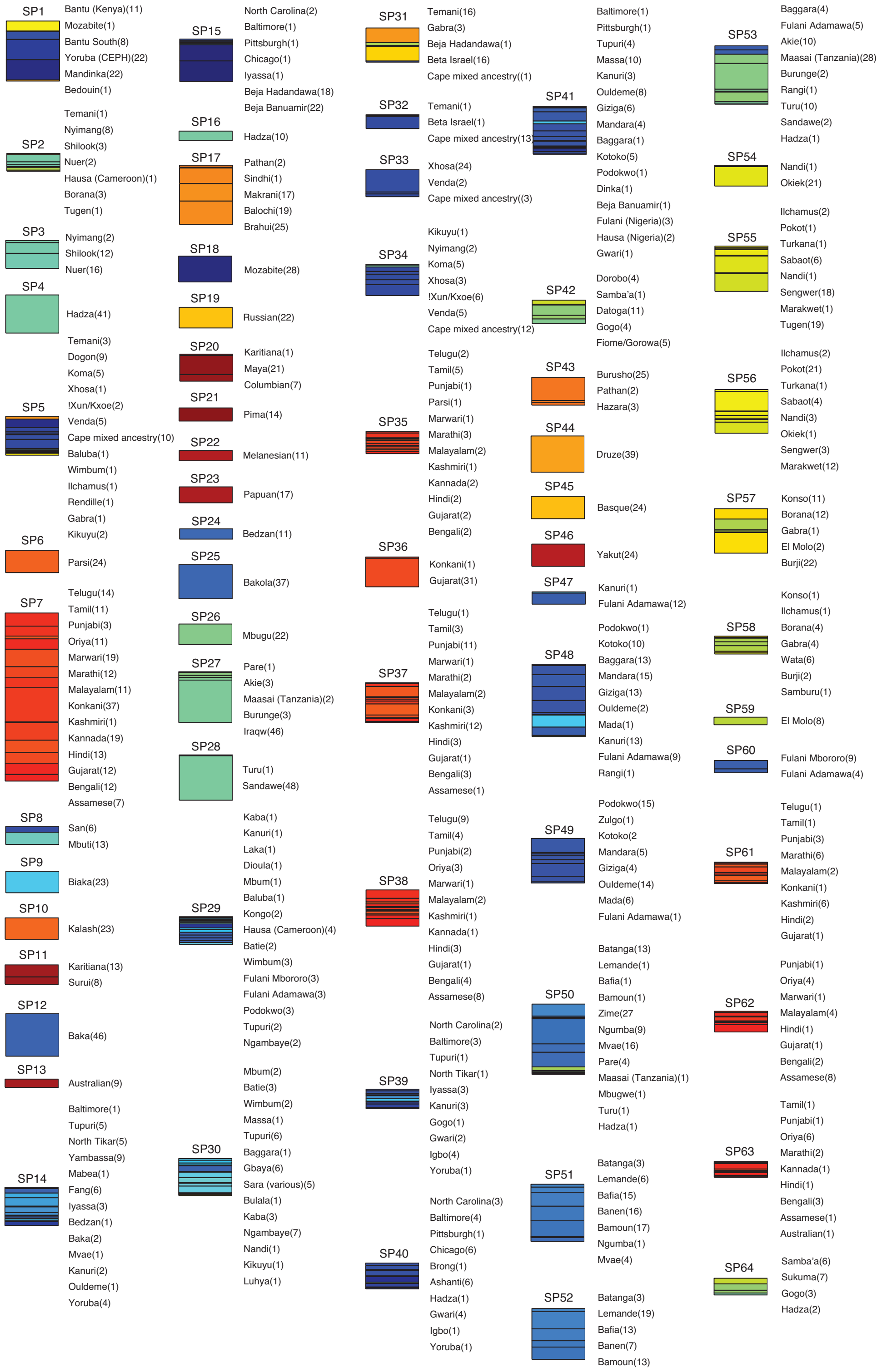
SP1
Bantu (Kenya)(11)
Mozabite(1)
Bantu South(8)
Yoruba (CEPH)(22)
Mandinka(22)
Bedouin(1)
SP2
Temani(1)
Nyimang(8)
Shilook(3)
Nuer(2)
Hausa (Cameroon)(1)
Borana(3)
Tugen(1)
SP3
Nyimang(2)
Shilook(12)
Nuer(16)
SP4
Hadza(41)
SP5
Temani(3)
Dogon(9)
Koma(5)
Xhosa(1)
!Xun/Kxoe(2)
Venda(5)
Cape mixed ancestry(10)
Baluba(1)
Wimbum(1)
Ilchamus(1)
Rendille(1)
Gabra(1)
Kikuyu(2)
SP6
Parsi(24)
SP7
Telugu(14)
Tamil(11)
Punjabi(3)
Oriya(11)
Marwari(19)
Marathi(12)
Malayalam(11)
Konkani(37)
Kashmiri(1)
Kannada(19)
Hindi(13)
Gujarat(12)
Bengali(12)
Assamese(7)
SP8
San(6)
Mbuti(13)
SP9
Biaka(23)
SP10
Kalash(23)
SP11
Karitiana(13)
Surui(8)
SP12
Baka(46)
SP13
Australian(9)
SP14
Baltimore(1)
Tupuri(5)
North Tikar(5)
Yambassa(9)
Mabea(1)
Fang(6)
Iyassa(3)
Bedzan(1)
Baka(2)
Mvae(1)
Kanuri(2)
Ouldeme(1)
Yoruba(4)
SP15
North Carolina(2)
Baltimore(1)
Pittsburgh(1)
Chicago(1)
Iyassa(1)
Beja Hadandawa(18)
Beja Banuamir(22)
SP16
Hadza(10)
SP17
Pathan(2)
Sindhi(1)
Makrani(17)
Balochi(19)
Brahui(25)
SP18
Mozabite(28)
SP19
Russian(22)
SP20
Karitiana(1)
Maya(21)
Columbian(7)
SP21
Pima(14)
SP22
Melanesian(11)
SP23
Papuan(17)
SP24
Bedzan(11)
SP25
Bakola(37)
SP26
Mbugu(22)
SP27
Pare(1)
Akie(3)
Maasai (Tanzania)(2)
Burunge(3)
Iraqw(46)
SP28
Turu(1)
Sandawe(48)
SP29
Kaba(1)
Kanuri(1)
Laka(1)
Dioula(1)
Mbum(1)
Baluba(1)
Kongo(2)
Hausa (Cameroon)(4)
Batie(2)
Wimbum(3)
Fulani Mbororo(3)
Fulani Adamawa(3)
Podokwo(3)
Tupuri(2)
Ngambaye(2)
SP30
Mbum(2)
Batie(3)
Wimbum(2)
Massa(1)
Tupuri(6)
Baggara(1)
Gbaya(6)
Sara (various)(5)
Bulala(1)
Kaba(3)
Ngambaye(7)
Nandi(1)
Kikuyu(1)
Luhya(1)
SP31
Temani(16)
Gabra(3)
Beja Hadandawa(1)
Beta Israel(16)
Cape mixed ancestry((1)
SP32
Temani(1)
Beta Israel(1)
Cape mixed ancestry(13
SP33
Xhosa(24)
Venda(2)
Cape mixed ancestry((3)
SP34
Kikuyu(1)
Nyimang(2)
Koma(5)
Xhosa(3)
!Xun/Kxoe(6)
Venda(5)
Cape mixed ancestry(12)
SP35
Telugu(2)
Tamil(5)
Punjabi(1)
Parsi(1)
Marwari(1)
Marathi(3)
Malayalam(2)
Kashmiri(1)
Kannada(2)
Hindi(2)
Gujarat(2)
Bengali(2)
SP36
Konkani(1)
Gujarat(31)
SP37
Telugu(1)
Tamil(3)
Punjabi(11)
Marwari(1)
Marathi(2)
Malayalam(2)
Konkani(3)
Kashmiri(12)
Hindi(3)
Gujarat(1)
Bengali(3)
Assamese(1)
SP38
Telugu(9)
Tamil(4)
Punjabi(2)
Oriya(3)
Marwari(1)
Malayalam(2)
Kashmiri(1)
Kannada(1)
Hindi(3)
Gujarat(1)
Bengali(4)
Assamese(8)
SP39
North Carolina(2)
Baltimore(3)
Tupuri(1)
North Tikar(1)
Iyassa(3)
Kanuri(3)
Gogo(1)
Gwari(2)
Igbo(4)
Yoruba(1)
SP40
North Carolina(3)
Baltimore(4)
Pittsburgh(1)
Chicago(6)
Brong(1)
Ashanti(6)
Hadza(1)
Gwari(4)
Igbo(1)
Yoruba(1)
SP41
Baltimore(1)
Pittsburgh(1)
Tupuri(4)
Massa(10)
Kanuri(3)
Ouldeme(8)
Giziga(6)
Mandara(4)
Baggara(1)
Kotoko(5)
Podokwo(1)
Dinka(1)
Beja Banuamir(1)
Fulani (Nigeria)(3)
Hausa (Nigeria)(2)
Gwari(1)
SP42
Dorobo(4)
Samba'a(1)
Datoga(11)
Gogo(4)
Fiome/Gorowa(5)
SP43
Burusho(25)
Pathan(2)
Hazara(3)
SP44
Druze(39)
SP45
Basque(24)
SP46
Yakut(24)
SP47
Kanuri(1)
Fulani Adamawa(12)
SP48
Podokwo(1)
Kotoko(10)
Baggara(13)
Mandara(15)
Giziga(13)
Ouldeme(2)
Mada(1)
Kanuri(13)
Fulani Adamawa(9)
Rangi(1)
SP49
Podokwo(15)
Zulgo(1)
Kotoko(2
Mandara(5)
Giziga(4)
Ouldeme(14)
Mada(6)
Fulani Adamawa(1)
SP50
Batanga(13)
Lemande(1)
Bafia(1)
Bamoun(1)
Zime(27
Ngumba(9)
Mvae(16)
Pare(4)
Maasai (Tanzania)(1)
Mbugwe(1)
Turu(1)
Hadza(1)
SP51
Batanga(3)
Lemande(6)
Bafia(15)
Banen(16)
Bamoun(17)
Ngumba(1)
Mvae(4)
SP52
Batanga(3)
Lemande(19)
Bafia(13)
Banen(7)
Bamoun(13)
SP53
Baggara(4)
Fulani Adamawa(5)
Akie(10)
Maasai (Tanzania)(28)
Burunge(2)
Rangi(1)
Turu(10)
Sandawe(2)
Hadza(1)
SP54
Nandi(1)
Okiek(21)
SP55
Ilchamus(2)
Pokot(1)
Turkana(1)
Sabaot(6)
Nandi(1)
Sengwer(18)
Marakwet(1)
Tugen(19)
SP56
Ilchamus(2)
Pokot(21)
Turkana(1)
Sabaot(4)
Nandi(3)
Okiek(1)
Sengwer(3)
Marakwet(12)
SP57
Konso(11)
Borana(12)
Gabra(1)
El Molo(2)
Burji(22)
SP58
Konso(1)
Ilchamus(1)
Borana(4)
Gabra(4)
Wata(6)
Burji(2)
Samburu(1)
SP59
El Molo(8)
SP60
Fulani Mbororo(9)
Fulani Adamawa(4)
SP61
Telugu(1)
Tamil(1)
Punjabi(3)
Marathi(6)
Malayalam(2)
Konkani(1)
Kashmiri(6)
Hindi(2)
Gujarat(1)
SP62
Punjabi(1)
Oriya(4)
Marwari(1)
Malayalam(4)
Hindi(1)
Gujarat(1)
Bengali(2)
Assamese(8)
SP63
Tamil(1)
Punjabi(1)
Oriya(6)
Marathi(2)
Kannada(1)
Hindi(1)
Bengali(3)
Assamese(1)
Australian(1)
SP64
Samba'a(6)
Sukuma(7)
Gogo(3)
Hadza(2)

Figure s1

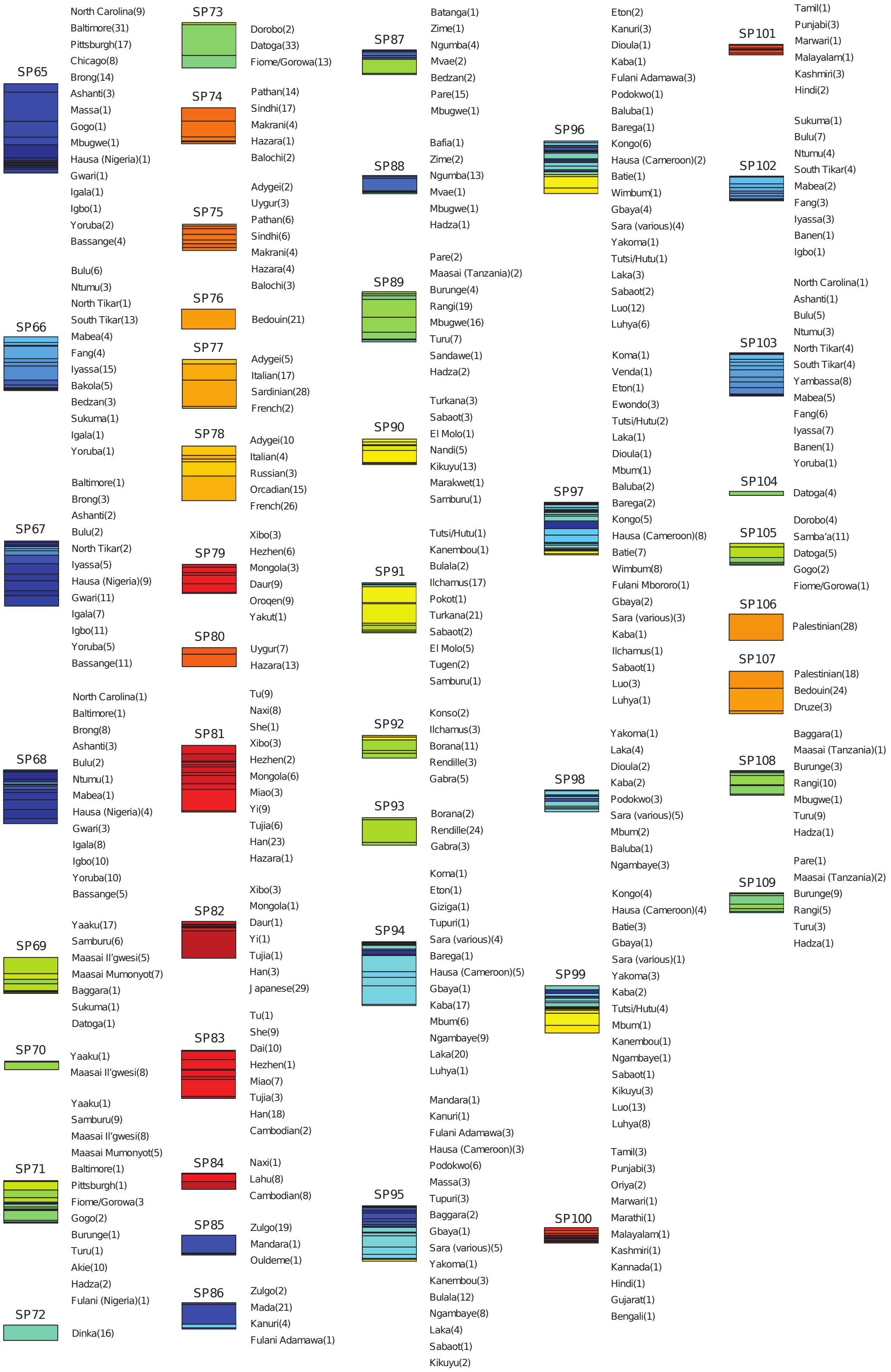
SP65
North Carolina(9)
Baltimore(31)
Pittsburgh(17)
Chicago(8)
Brong(14)
Ashanti(3)
Massa(1)
Gogo(1)
Mbugwe(1)
Hausa (Nigeria)(1)
Gwari(1)
Igala(1)
Igbo(1)
Yoruba(2)
Bassange(4)
SP66
Bulu(6)
Ntumu(3)
North Tikar(1)
South Tikar(13)
Mabea(4)
Fang(4)
Iyassa(15)
Bakola(5)
Bedzan(3)
Sukuma(1)
Igala(1)
Yoruba(1)
SP67
Baltimore(1)
Brong(3)
Ashanti(2)
Bulu(2)
North Tikar(2)
Iyassa(5)
Hausa (Nigeria)(9)
Gwari(11)
Igala(7)
Igbo(11)
Yoruba(5)
Bassange(11)
SP68
North Carolina(1)
Baltimore(1)
Brong(8)
Ashanti(3)
Bulu(2)
Ntumu(1)
Mabea(1)
Hausa (Nigeria)(4)
Gwari(3)
Igala(8)
Igbo(10)
Yoruba(10)
Bassange(5)
SP69
Yaaku(17)
Samburu(6)
Maasai Il'gwesi(5)
Maasai Mumonyot(7)
Baggara(1)
Sukuma(1)
Datoga(1)
SP70
Yaaku(1)
Maasai Il'gwesi(8)
SP71
Yaaku(1)
Samburu(9)
Maasai Il'gwesi(8)
Maasai Mumonyot(5)
Baltimore(1)
Pittsburgh(1)
Fiome/Gorowa(3
Gogo(2)
Burunge(1)
Turu(1)
Akie(10)
Hadza(2)
Fulani (Nigeria)(1)
SP72
Dinka(16)
SP73
Dorobo(2)
Datoga(33)
Fiome/Gorowa(13)
SP74
Pathan(14)
Sindhi(17)
Makrani(4)
Hazara(1)
Balochi(2)
SP75
Adygei(2)
Uygur(3)
Pathan(6)
Sindhi(6)
Makrani(4)
Hazara(4)
Balochi(3)
SP76
Bedouin(21)
SP77
Adygei(5)
Italian(17)
Sardinian(28)
French(2)
SP78
Adygei(10
Italian(4)
Russian(3)
Orcadian(15)
French(26)
SP79
Xibo(3)
Hezhen(6)
Mongola(3)
Daur(9)
Oroqen(9)
Yakut(1)
SP80
Uygur(7)
Hazara(13)
SP81
Tu(9)
Naxi(8)
She(1)
Xibo(3)
Hezhen(2)
Mongola(6)
Miao(3)
Yi(9)
Tujia(6)
Han(23)
Hazara(1)
SP82
Xibo(3)
Mongola(1)
Daur(1)
Yi(1)
Tujia(1)
Han(3)
Japanese(29)
SP83
Tu(1)
She(9)
Dai(10)
Hezhen(1)
Miao(7)
Tujia(3)
Han(18)
Cambodian(2)
SP84
Naxi(1)
Lahu(8)
Cambodian(8)
SP85
Zulgo(19)
Mandara(1)
Ouldeme(1)
SP86
Zulgo(2)
Mada(21)
Kanuri(4)
Fulani Adamawa(1)
SP87
Batanga(1)
Zime(1)
Ngumba(4)
Mvae(2)
Bedzan(2)
Pare(15)
Mbugwe(1)
SP88
Bafia(1)
Zime(2)
Ngumba(13)
Mvae(1)
Mbugwe(1)
Hadza(1)
SP89
Pare(2)
Maasai (Tanzania)(2)
Burunge(4)
Rangi(19)
Mbugwe(16)
Turu(7)
Sandawe(1)
Hadza(2)
SP90
Turkana(3)
Sabaot(3)
El Molo(1)
Nandi(5)
Kikuyu(13)
Marakwet(1)
Samburu(1)
SP91
Tutsi/Hutu(1)
Kanembou(1)
Bulala(2)
Ilchamus(17)
Pokot(1)
Turkana(21)
Sabaot(2)
El Molo(5)
Tugen(2)
Samburu(1)
SP92
Konso(2)
Ilchamus(3)
Borana(11)
Rendille(3)
Gabra(5)
SP93
Borana(2)
Rendille(24)
Gabra(3)
SP94
Koma(1)
Eton(1)
Giziga(1)
Tupuri(1)
Sara (various)(4)
Barega(1)
Hausa (Cameroon)(5)
Gbaya(1)
Kaba(17)
Mbum(6)
Ngambaye(9)
Laka(20)
Luhya(1)
SP95
Mandara(1)
Kanuri(1)
Fulani Adamawa(3)
Hausa (Cameroon)(3)
Podokwo(6)
Massa(3)
Tupuri(3)
Baggara(2)
Gbaya(1)
Sara (various)(5)
Yakoma(1)
Kanembou(3)
Bulala(12)
Ngambaye(8)
Laka(4)
Sabaot(1)
Kikuyu(2)
SP96
Eton(2)
Kanuri(3)
Dioula(1)
Kaba(1)
Fulani Adamawa(3)
Podokwo(1)
Baluba(1)
Barega(1)
Kongo(6)
Hausa (Cameroon)(2)
Batie(1)
Wimbum(1)
Gbaya(4)
Sara (various)(4)
Yakoma(1)
Tutsi/Hutu(1)
Laka(3)
Sabaot(2)
Luo(12)
Luhya(6)
SP97
Koma(1)
Venda(1)
Eton(1)
Ewondo(3)
Tutsi/Hutu(2)
Laka(1)
Dioula(1)
Mbum(1)
Baluba(2)
Barega(2)
Kongo(5)
Hausa (Cameroon)(8)
Batie(7)
Wimbum(8)
Fulani Mbororo(1)
Gbaya(2)
Sara (various)(3)
Kaba(1)
Ilchamus(1)
Sabaot(1)
Luo(3)
Luhya(1)
SP98
Yakoma(1)
Laka(4)
Dioula(2)
Kaba(2)
Podokwo(3)
Sara (various)(5)
Mbum(2)
Baluba(1)
Ngambaye(3)
SP99
Kongo(4)
Hausa (Cameroon)(4)
Batie(3)
Gbaya(1)
Sara (various)(1)
Yakoma(3)
Kaba(2)
Tutsi/Hutu(4)
Mbum(1)
Kanembou(1)
Ngambaye(1)
Sabaot(1)
Kikuyu(3)
Luo(13)
Luhya(8)
SP100
Tamil(3)
Punjabi(3)
Oriya(2)
Marwari(1)
Marathi(1)
Malayalam(1)
Kashmiri(1)
Kannada(1)
Hindi(1)
Gujarat(1)
Bengali(1)
SP101
Tamil(1)
Punjabi(3)
Marwari(1)
Malayalam(1)
Kashmiri(3)
Hindi(2)
SP102
Sukuma(1)
Bulu(7)
Ntumu(4)
South Tikar(4)
Mabea(2)
Fang(3)
Iyassa(3)
Banen(1)
Igbo(1)
SP103
North Carolina(1)
Ashanti(1)
Bulu(5)
Ntumu(3)
North Tikar(4)
South Tikar(4)
Yambassa(8)
Mabea(5)
Fang(6)
Iyassa(7)
Banen(1)
Yoruba(1)
SP104
Datoga(4)
SP105
Dorobo(4)
Samba'a(11)
Datoga(5)
Gogo(2)
Fiome/Gorowa(1)
SP106
Palestinian(28)
SP107
Palestinian(18)
Bedouin(24)
Druze(3)
SP108
Baggara(1)
Maasai (Tanzania)(1)
Burunge(3)
Rangi(10)
Mbugwe(1)
Turu(9)
Hadza(1)
SP109
Pare(1)
Maasai (Tanzania)(2)
Burunge(9)
Rangi(5)
Turu(3)
Hadza(1)

Figure s2

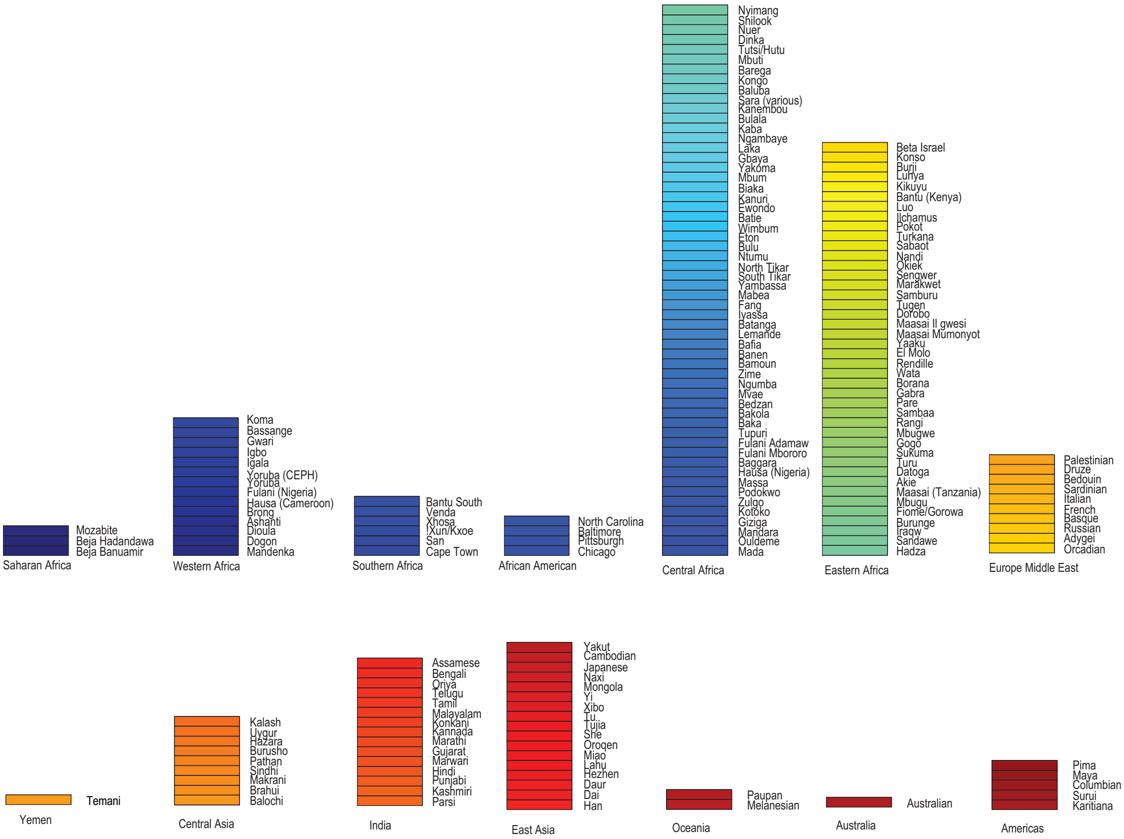

Figure s3

References

1. Tishkoff SA, Reed FA, Friedlaender FR, Ehret C, Ranciaro A, Froment A, Hirbo JB, Awomoyi AA, Bodo JM, Doumbo O, Ibrahim M, Juma AT, Kotze MJ, Lema G, Moore JH, Mortensen H, Nyambo TB, Omar SA, Powell K, Pretorius GS, Smith MW, Thera MA, Wambebe C, Weber JL, Williams SM: **The genetic structure and history of Africans and African Americans**. *Science* 2009, **324**(5930):1035–44.

2. Campbell MC, Tishkoff SA: **The evolution of human genetic and phenotypic variation in Africa**. *Curr Biol* 2010, **20**(4):R166–73.

3. Marlowe F: **Mate preferences among Hadza hunter-gatherers**. *Human Nature* 2004, **15**:365–376.
